# Differences in referral rates to specialised health care from four primary health care models in Klaipeda, Lithuania

**DOI:** 10.1186/1471-2296-9-63

**Published:** 2008-11-26

**Authors:** Andrzej Zielinski, Anders Håkansson, Arnoldas Jurgutis, Ingvar Ovhed, Anders Halling

**Affiliations:** 1 Lund University, Department of Clinical Sciences in Malmö, General Practice/Family Medicine, SE-205 02, Malmö, Sweden; 2 Blekinge Competence Centre, Wämö Centre, SE-371 81, Karlskrona, Sweden; 3 Public Health Department, Klaipeda University, Klaipeda, Lithuania

## Abstract

**Background:**

Lithuanian primary health care (PHC) is undergoing changes from the systems prevalent under the Soviet Union, which ensured free access to specialised health care. Currently four different PHC models work in parallel, which offers the opportunity to study their respective effect on referral rates. Our aim was to investigate whether there were differences in referrals rates from different Lithuanian PHC models in Klaipeda after adjustment for co-morbidity.

**Methods:**

The population listed with 18 PHC practices serving inhabitants in Klaipeda city and region (250 070 inhabitants). Four PHC models: rural state-owned family medicine practices, urban privately owned family medicine practices, state-owned polyclinics and privately owned polyclinics. Information on listed patients and referrals during 2005 from each PHC practice in Klaipeda was obtained from the Lithuanian State Sickness Fund database. The database records included information on age, gender, PHC model, referrals and ICD 10 diagnoses. The Johns Hopkins ACG Case-Mix system was used to study co-morbidity. Referral rates from different PHC models were studied using Poisson regression models.

**Results:**

Patients listed with rural state-owned family medicine practices had a significantly lower referral rate to specialised health care than those in the other three PHC models. An increasing co-morbidity level correlated with a higher physician- to self-referral ratio.

**Conclusion:**

Family medicine practices located in rural-, but not in urban areas had significantly lower referral rates to specialised health care. It could not be established whether this was due to organisation, training of physicians or financing, but suggests there is room for improving primary health care in urban areas. Patient's place of residence and co morbidity level were the most important factors for referral rate. We also found that gatekeeping had an effect on the referral pattern with respect to co-morbidity level, so that those with a physician referral were more likely to have had higher co-morbidity.

## Background

Gatekeeping and referrals are important tasks of primary health care (PHC) in most countries [1,2]. The role of gatekeeping with a referral system ensures that other parts of the health-care system are able to specialise in different diseases or procedures. Gatekeeping has also been shown to decrease the number of medical procedures, specialist encounters and hospitalisations [1,3]. Moreover, increasing the number of specialists does not improve a population's health [4]. Where specialised health care is more readily available, as in the USA, it is an important factor that influences referral rates [5-7]. When gatekeeping was introduced in some states in the USA, the number of specialist referrals and the consumption of specialised health care decreased [1].

In Sweden, team-based PHC, centred around PHC physicians who work as generalists, has been developed since the 1970s. This resulted in comparatively low referral rates to specialised health care and a decrease in utilisation and costs [8].

Currently, there is a move to transfer specialists into PHC (närsjukvård) in Sweden due to a shortage of specialists in family medicine.

The Lithuanian PHC system is undergoing changes from the Soviet Union system, which offered free access to specialised health care, to a system in which the PHC physicians act as gatekeepers [9]. Since 1997–1998, after the introduction of the Lithuanian State Sickness Fund, patients have been listed to specified PHC practices [9]. Currently, PHC is provided both by family medicine practices and in polyclinics, where PHC physicians work with secondary health care specialists. Both family medicine practices and polyclinics can be privately or state-owned.

Thus, four different PHC models work in parallel in Lithuania today. This allows us to compare their respective effect on referrals, which could also be of importance for other countries. We hypothesised that family medicine practices would provide more comprehensive [10] PHC services than the polyclinics, which were the only PHC providers before the health care reform in 1997.

No previous studies on referrals in the former Eastern Europe have been done after taking co-morbidity into account.

Our aim was to study if there were lower referral rates to specialised health care in patients listed to the recently introduced family medicine practices.

## Methods

### Study population

The population listed with 18 PHC practices, serving 250,070 inhabitants in Klaipeda city and the Klaipeda region, was included (Table 1). Data on the population listed at PHC practices in Klaipeda city and region during 2005 were obtained from the Lithuanian State Sickness Fund database. The Sickness Fund reimbursed both state- and privately owned PHC practices for their services using information in this database, which ensured that the validity of the data was high. The database records included information on age, gender, PHC practices, referrals and ICD 10 diagnoses.

**Table 1:** Characteristics of population and variables included (I).

	**Public practices**	**Private practices**	**Public polyclinics**	**Private polyclinics**
	**N (%)**	**N (%)**	**N (%)**	**N (%)**
	
Number of listed patients	10973	41849	173738	23510
				
***Gender***				
female	5663 (51.61%)	23275 (55.62%)	90839 (52.28%)	13480 (57.34%)
male	5310 (48.39%)	18574 (44.38%)	82899 (47.72%)	10030 (42.66%)
***Age***				
0–19	3204 (29.20%)	12509 (29.89%)	31415 (18.08%)	7841 (33.35%)
20–39	3178 (28.96%)	12266 (29.31%)	55598 (32.00%)	7486 (31.84%)
40–59	2511 (22.88%)	10177 (24.32%)	51362 (29.56%)	5006 (21.29%)
60–79	1787 (16.28%)	5789 (13.83%)	31011 (17.85%)	2716 (11.55%)
over 80	293 (2.67%)	1108 (2.65%)	4352 (2.50%)	461 (1.96%)
***Place of residence***				
urban	3076 (28.03%)	38423 (91.81%)	158081 (90.99%)	23126 (98.37%)
rural	7897 (71.97%)	3426 (8.19%)	15657 (9.01%)	384 (1.63%)
***Co-morbidity level***				
RUB 0	2963 (27.00%)	10858 (25.94%)	60983 (35.10%)	4998 (21.26%)
RUB 1	2744 (25.01%)	7057 (16.83%)	25429 (14.64%)	4264 (18.14%)
RUB 2	2383 (21.72%)	10558 (25.23%)	35468 (20.41%)	6331 (26.93%)
RUB 3	2542 (23.16%)	11776 (28.14%)	45147 (25.98%)	6952 (29.57%)
RUB 4	301 (2.74%)	1394 (3.33%)	5794 (3.33%)	846 (3.60%)
RUB 5	40 (0.36%)	206 (0.49%)	917 (0.53%)	119 (0.51%)

RUB – resource utilisation band

This study was performed according to Lithuanian law, and the data obtained from the Sickness Fund used encrypted identification numbers, which ensured the anonymity of the participants.

### Dependent variable

The dependent variable was the number and type (physician/self) of referrals to specialised health care per 1000 patient years, according to which PHC practice in the Klaipeda city/region at which the patient was listed during 2005. Information on the type of referral was sent to the Lithuanian State Sickness Fund by the specialist together with other information about the visit. Referrals included all the registered specialist referrals in 2005. The physician referrals were both from PHC physicians and from specialists to specialists, as it was not possible to separate them. Patients in Lithuania need a referral to specialists to avoid paying for the consultation. However, it is also possible for patients to meet a specialist without a referral from a PHC physician, but they then have to pay per visit.

### Independent variables

#### 1. Model of primary health care

All the 18 PHC practices were divided into four groups, depending on the availability of specialised health care at the practice and type of ownership:

1. PHC practices which provided only PHC services.

a) State-owned family medicine practices (public practices) located in a rural area, with 100% family physicians working at the practice (Table 2). The practices had no direct access to specialised health care and provided only PHC services. Most of the listed patients lived in a rural area (Table 1).

**Table 2:** PHC and physician characteristics in the four PHC models

	**PHC model**		**Physicians in PHC**	
	**N**	**Rural (%)**	**N**	**Fam. med. spec. (%)**

Public practices	3	100	9	100.0
Private practices	8	25	40	72.5
Public polyclinics	4	25	144	48.6
Private polyclinics	3	0	23	73.9

b) Privately owned family medicine practices (private practices) mostly located in urban areas (75%), in which the majority of the physicians working at the practice were trained as family physicians (Table 2). The other physicians also worked as family physicians without having formal training. These practices had no direct access to specialised health care and provided only PHC services. Most of its patients lived in urban areas (Table 1).

2. PHC practices which to some extent combined PHC and secondary health care services (polyclinics).

a) State-owned practices of the polyclinic type (public polyclinics), mostly located in urban areas (75%), with family physicians working together with different specialists at the same location (Table 2). Most of the listed patients lived in urban areas (Table 1).

b) Privately owned practices of the polyclinic type (private polyclinics), located in urban areas, with family physicians working together with different specialists at the same location (Table 2). These practices provided both PHC and secondary health care services. Most of the listed patients lived in urban areas (Table 1).

#### 2. Co-morbidity

The Johns Hopkins ACG (Adjusted Clinical Groups) Case-Mix System [11] was used as a measure of co-morbidity. This system was developed in the 1980s to evaluate the relationship between patient morbidity and utilisation of health care services. The system is based on the theory that co-morbidity corresponds to a certain need for health care resources. The ACG Case-Mix System groups patients to one of 82 ACG levels, which depend on the types of morbidity that are characterised by five criteria: 1. Likely persistence of the condition. 2. Severity of the condition. 3. Aetiology. 4. Diagnostic certainty. 5. Need for specialised health care. Each ACG group consists of patients with the same type and degree of co-morbidity [12-14]. Diagnoses were obtained from the Lithuanian Sickness Fund database. Data were based on information from all PHC and secondary health care providers in Klaipeda region during year 2005. Diagnoses were then grouped using the ACG Case-Mix System 7.1. In our study all patients in the study population were assigned to one of six levels of co-morbidity, resource utilisation bands (RUBs). The population in RUB 0 had no need of health care, whereas those in RUB 5 had a very high need of health care resources.

#### 3. Age

#### 4. Gender

#### 5. Patient's place of residence (rural/urban)

Urban patients were living in the three cities of the study area: Klaipeda, Gargzdai and Paupai.

Rural patients were living in Klaipeda region outside the administrative areas of these three cities.

### Statistical analysis

The referral rates from four different PHC models where studied using Poisson regression models [15], adjusting for clustering on the level of PHC practice (STATA version 10, Stata Corporation, Texas, USA). We analysed physician- and self-referrals separately. Physician- and self-referrals were adjusted for co-variables: PHC model, age, gender, patient's place of residence (rural/urban) and RUB. Physician- and self-referrals were adjusted with the covariables, which were successively introduced and estimated in four models. Model A presented referral ratios from the different PHC models univariately; in model B the referral ratios were adjusted for age and gender. In model C, the patient's place of residence was added, and in model D all co-variables were included (also including co-morbidity).

## Results

### Referral rates

The three public practices served mainly rural populations and were staffed entirely by family physicians (Table 2).

During the year of the study, 69% of all visits were to primary care physicians. The overall proportion of the listed population who visited a specialist in the year was 43.4% (Table 3). Public practices had significantly lower referral rates for both physician- and self-referrals than the other three PHC models although the proportion of physician- to self-referrals was similar across the four PHC types. Urban practices had higher rates of referral for both physician- and self-referrals (Table 4).

**Table 3:** Proportion of all listed patients who saw a specialist in year 2005

**Included variables**	**Proportion of all listed patients who met specialist during 2005 (%)**
**All**	**43.43**
***PHC model***	
Public practices	34.8
Private practices	47.71
Public polyclinics	41.74
Private polyclinics	51.65
***Age-years***	
0–19	54.76
20–39	33.18
40–59	39.55
60–79	54.14
80-	45.30
***Gender***	
female	46.17
male	40.30
***Patient's place of residence***	
urban	44.03
rural	38.22
***Co-morbidity level (RUB)***	
RUB 0	16.48
RUB 1	39.81
RUB 2	63.70
RUB 3	72.94
RUB 4	89.90
RUB 5	94.94

RUB – resource utilisation band

**Table 4:** Characteristics of population and variables included (II).

			**Physician referrals**		**Self-referrals**		
				
**Included variables**	**Number of individuals**	**Proportion of all listed patients**	**Referrals/1000 patient years**	**Proportion of referrals/1000 patient years**	**Referrals/1000 patient years**	**Proportion of referrals/1000 patient years**	**Proportion of self-referrals to all referrals**
	**N**	**%**	**N**	**%**	**N**	**%**	**%**
PHC model							
Public practices	10973	4.4	780	17.6	174	14.9	18.3
Private practices	41849	16.7	1271	28.6	335	28.8	20.9
Public polyclinics	173738	69.5	1114	25.1	309	26.5	21.7
Private polyclinics	23510	9.4	1278	28.8	346	29.7	21.3
Age-years							
0–19	54969	22.0	1348	21.5	290	17.8	17.7
20–39	78528	31.4	633	10.1	322	19.7	33.7
40–59	69056	27.6	1075	17.1	286	17.5	21.0
60–79	41303	16.5	1922	30.6	377	23.1	16.4
80-	6214	2.5	1296	20.7	355	21.8	21.5
Gender							
female	133257	53.3	1269	56.0	321	51.4	20.2
male	116813	46.7	996	44.0	304	48.6	23.4
Patient's place of residence							
urban	222706	89.1	1166	55.4	331	66.3	22.1
rural	27364	10.9	940	44.6	168	33.7	15.2
Co-morbidity level (RUB)							
RUB 0	79802	31.9	2	0.01	2	0.1	50.0
RUB 1	39494	15.8	254	1.5	196	7.7	43.6
RUB 2	54740	21.9	1113	6.6	448	17.6	28.7
RUB 3	66417	26.6	2423	14.4	543	21.4	18.3
RUB 4	8335	3.3	5214	31.0	959	37.8	15.5
RUB 5	1282	0.5	7797	46.4	392	15.4	4.8

RUB – resource utilisation band

**Figure 1:**
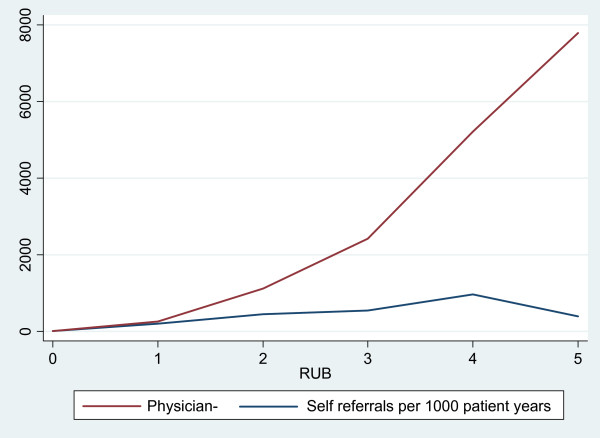
**Physician- and self-referrals per 1000 patient years according to increased comorbidity level**. RUB – resource utilisation band

Referral rates were higher for female patients for both physician- (21.5%) and self-referral (5.3%). The proportion of self-referrals was also higher in patients of ages 20–39 and patients living in urban areas. The rate of physician referrals increased proportionally with co-morbidity level, but a higher level of co-morbidity was correlated with a significantly lower probability of self-referral (Table 4) as the physician referral rates increased markedly with increasing co-morbidity but this was not the case for self-referrals (Figure 1).

The highest rate of both physician- and self-referrals was in the age group 60–79 years (Table 4).

### Correlates of physician referral

To clarify the differences across the four PHC models, we adjusted for the patient's age and gender, place of residence (rural/urban), and co-morbidity level in four successive models. Table 5 shows that the referral ratio was 42–63% lower in the public practices than in the three other PHC models. The most important factor influencing referral rates from private practices and polyclinics, which could be seen by a reduction in incidence rate ratio by 23 and 34% respectively was co-morbidity. However, even after adjusting for age, gender, patient's place of residence and co-morbidity, the difference between the public practices model and the other was still 19–28% (Table 5).

**Table 5:** Physician referral rates to specialists from different PHC practice models adjusted for patient's age, gender, patient's place of residence (urban/rural) and co-morbidity level.

**Included variables**	**Referrals/1000 patient years**	**Model A**		**Model B**		**Model C**		**Model D**	
	**N**	**IRR**	**95%CI**	**IRR**	**95%CI**	**IRR**	**95%CI**	**IRR**	**95%CI**
	
Public practices	780	1.0		1.0		1.0		1.0	
Private practices	1271	1.63***	1.41–1.88	1.63***	1.39–1.91	1.51***	1.28–1.78	1.28**	1.07–1.53
Public polyclinics	1114	1.42***	1.22–1.66	1.38***	1.16–1.64	1.28**	1.10–1.49	1.27**	1.06–1.51
Private polyclinics	1278	1.63***	1.38–1.93	1.67***	1.39–1.99	1.53***	1.26–1.85	1.19	0.98–1.45

* p < .05 ** p < .01 *** p < .001

IRR – incidence rate ratio, CI – confidence interval, RUB – resource utilisation band, PHC – primary health care model

Model A: unadjusted

Model B: adjusted for age and gender

Model C: adjusted for age and gender and urban/rural

Model D: adjusted for age and gender, urban/rural and level of co-morbidity (RUB)

### Self-referrals

There was an even larger difference in referral ratios between the public practices model and the other PHC models, than was shown for physician referrals in the unadjusted model. This difference was completely explained by the patient's place of residence. Adjusting for co-morbidity level did not have any further effect (Table 6).

**Table 6:** Self-referral rates to specialist from different PHC practice models adjusted for patient's age, gender, patient's place of residence (urban/rural) and co-morbidity level.

**Included variables**	**Referrals/1000 patient years**	**Model A**		**Model B**		**Model C**		**Model D**	
	**N**	**IRR**	**95%CI**	**IRR**	**95%CI**	**IRR**	**95%CI**	**IRR**	**95%CI**
	
Public practices	174	1.0		1.0		1.0		1.0	
Private practices	335	1.92***	1.40–2.63	1.92***	1.39–2.65	1.35	0.94–1.93	1.20	0.82–1.75
Public polyclinics	309	1.77***	1.25–2.52	1.75**	1.23–2.50	1.23	0.83–1.82	1.27	0.82–1.95
Private polyclinics	346	2.08***	1.52–2.86	2.10***	1.52–2.90	1.43	0.99–2.07	1.19	0.81–1.75

* p < .05 ** p < .01 *** p < .001

IRR – incidence rate ratio, CI – confidence interval, RUB – resource utilisation band, PHC – primary health care model

Model A: unadjusted

Model B: adjusted for age and gender

Model C: adjusted for age and gender and urban/rural

Model D: adjusted for age and gender, urban/rural and level of co-morbidity(RUB)

## Discussion

### The main findings

This study shows that the referral rate in patients listed at family medicine practices in rural areas but not in urban areas was lower than in patients listed at the other PHC models, even after adjusting for the patient's age, gender, place of residence and co-morbidity. The most important factors that influenced referral rates were the patient's place of residence (rural/urban) and level of co-morbidity. Patients living in urban areas and with higher co-morbidity level were referred by physicians to specialists more frequently (Table 4). In the model, age and gender had only minor effects (Tables 5 and 6).

### Previous work

Case-mix, age, gender, and co-morbidity, patient's demands, and physician's and organisational characteristics of the health care system (gatekeeping, specialist supply) influence both physician- and self-referrals [12,15-18].

Patient's characteristics explain about 40% of the variability in referral rates whereas facility characteristics and family medicine physicians explain about 10% [15]. Our study showed the type of PHC model, physician's training, PHC practice location, patient's place of residence (rural/urban), and co-morbidity also are major influences on referrals. Mode of paying for visits might also have been important but we did not have the data to examine this. There is some evidence to suggest that the way in which a PHC physician is paid affects their behaviour [19].

### PHC practice model

The progress of change in the Lithuanian PHC system is slow, and most people (70%) who live in Klaipeda and the Klaipeda region are still listed with Soviet-type polyclinics (public polyclinics)(Table 1, third column). Only about one-fifth of the population studied are listed with family medicine practices (public- and private practices)(Table 4, second column). Patients who are accustomed to easy access to specialist treatment in large polyclinics, especially in cities, may find it difficult to accept a new PHC system in which the family physician is the main, and often only, caregiver. Once referred, some patients continue their contact with specialists, especially if they have been satisfied with their previous consultations [20].

The per capita rate of specialist consultations is twice as high in the USA than in England. Furthermore, the rate of self-referrals in the USA is much higher than in England, where PHC forms the base of the health care system and gatekeeping is accepted [6]. The high rate of specialist supply in the USA correlates with both higher referral rates and patient expectations to be referred to a specialist [5,7]. This agrees with our results from Lithuania, where both the physician- and self-referral rates from public and private polyclinics (which have easier access to specialists) are higher than from public practices. Contrary to our hypothesis that family medicine practices lowered the referral rate, we found that this was not true in the urban areas. Referral rates from private practices were higher than from public practices and even higher than from public polyclinics. It suggests there is room for improving PHC in Lithuania in the urban areas. The reimbursement system, closeness to specialists and patient demands, might be important factors influencing the difference between rural and urban family medicine practices.

### Physician training

Physician characteristics such as being a specialist in family medicine, training and experience correlate with lower referral rates [21,22]. Family medicine as practised in public and private practices is quite a new element of the health care system in Lithuania. Physicians who work in these practices have completed their training in family medicine. They are therefore able to treat a vast range of common diseases and work independently of the hospital-based system. In polyclinics, where different types of specialists work in the same building as PHC physicians, earlier collaboration patterns with specialists can influence the referral rates [23]. This, however, does not explain the difference in referral rates between public practices and private practices providing only PHC and where most of physicians are specialists in family medicine.

### Patient's place of residence and PHC location

The main difference between public- and private PHC practices was geographic distance from specialists, which could influence the lower referral rate to specialists from public practices. Another difference might be that patients living in rural areas are more accustomed to visit to their PHC physicians, as their choise of physicians is smaller and it is the nearest physician they have. Some studies have shown that PHC physicians working in rural practices or smaller towns refer less frequently than PHC physicians who work in bigger towns with closer access to specialised health care [16,24,25]. This might explain why the referral rates of the other three PHC models did not differ in spite of organisational differences; nearly all of them were situated in towns or had close co-operation with specialists. The difference seen in self-referral rates between public practices and the other PHC models was fully explained by the patient's place of residence. This factor was also the most important for physician referral rates from the public polyclinics.

### Co-morbidity

Co-morbidity is a very important factor in explaining the rate of specialist referrals [26-28]. In our study we found that an increasing co-morbidity level was correlated with proportionally higher physician- to self-referral rate. Patients with chronic diseases seem to prefer visiting their family physician before being referred [29]. On the other hand, if they have an unstable chronic condition, they might prefer self-referral as it might shorten waiting time [20].

Co-morbidity was the most important factor for explaining physician referral rates in all PHC models except for the public polyclinic model, in which the referral rate was more influenced by patient's place of residence.

### Financing

The PHC practices in Lithuania that receive financing from the State Sickness Fund have a capitation system. PHC funding is not dependent on the amount of secondary health care utilisation. This reimbursement system creates little incentive for increases in productivity in PHC. Moreover, secondary health care is financed fee-for-service. Thus in the polyclinics this reimbursement system creates strong incentives to increase referrals and demand for secondary care in only slightly complicated cases [9]. A fee-for-service system can encourage higher utilisation of secondary health care than a capitation system [30].

### Discussion of method

The data received from the Lithuanian State Sickness Fund was our only source of information. Our results were limited by the data and we could not study other factors which might have influenced referral rates.

The quality of data according to the State Sickness Fund was high because the data is used as the base for reimbursement.

Data include information about physician referrals but it was impossible to divide them according to referrals from PHC physicians to specialists and from specialists to the other specialists. As a result, referral rates described the frequency of referrals in a particular PHC area but not the frequency of referrals from PHC. Thus we were not able to discern if the increased referral rates seen in urban PHC's were due to increased PHC to specialist referrals or specialist to specialist referrals.

## Conclusion

This study shows that the population listed to family medicine practices located in rural areas are less likely to be referred (adjusted for age, gender, co-morbidity and place of living). The rural family medicine practices seem to play the most effective role as gatekeepers in the Lithuanian health care system. Family medicine practices located in urban areas did not have lower referral rates, which agrees with other studies [14,23]. It is unclear why referral rates differ between rural and urban family medicine practices. Further studies of the influence of closeness to specialist and hospital, patients' demands and financing may provide an answer to this.

## Competing interests

The authors declare that they have no competing interests.

## Authors' contributions

AZ conceived the idea and designed the study, collected all the data and wrote the manuscript. AHÅ participated in the design of the study, helped to co-ordinate the work and drafted the manuscript. AJ helped to collect all the data and supplied information on the Lithuanian health care system. IO participated in the design of the study, drafted the manuscript and helped to co-ordinate the work. AHA supervised AZ, designed the study, wrote the manuscript, handled the ACG software, performed the statistical analyses, and coordinated the work. All authors read and approved the final manuscript.

## Pre-publication history

The pre-publication history for this paper can be accessed here:


